# Valedictory Address

**Published:** 1849-04

**Authors:** 


### VALEDICTORY ADDRESS.

BY DR. TAYLOR.

The fourth commencement exercises of the Ohio College of Dental Surgery, were held on the evening of the 19th February, 1849. After the conferring of the degrees, and an admirable address to the graduating class, by the President of the Board of Trustees, (Rev. Dr. Aydelotte,) the valedictory to the class was delivered by ourself. This, although hastily written at the close of the session, when pressed with other duties, yet, in accordance with the desire of the class, made known by the subjoined note, we consent to publish.—J. T.

Dental College,
February, 20, 1849.

Prof. Taylor:

Sir:—The dental class of the Ohio College of Dental Surgery have authorized us to request of you for publication in the Dental Register a copy of your valuable address, as delivered at the College on Monday evening, the 19th instant.

Yours, most respectfully,

T. R. WILLARD,

JNO. L. MILTON,

N. T. PRESTLEY, } *Committee.*

Gentlemen:—It would appear entirely unnecessary for me to add any thing to that which has already been said—custom, however, sanctions a valedictory address from the Dean or some member of the Faculty, on occasions like the present. It has always been to me an affecting scene to *even* witness the formal separation of friends—to see those who for months or years have been drawn together by social and relative duties, say farewell. It strikes a chord which vibrates on the past, and sends, in sympathetic unison, a thrill of irrepressible emotion to my heart. How much more, then, should an occasion like the present arouse such feelings, and excite in our minds a lively sense of that mutual good feeling which has sprung up and been caused by the kind manner, at *least* on *your part*, by which our relative duties have been reciprocated during the session now drawn to a close.

Permit me, however, more specially to address you in the name of the Faculty, and say, that your individual kindness, your untiring industry and close attention to study, has impressed on our minds the most grateful sense of your worth, and far more than any pecuniary consideration we could expect, remunerates us for our feeble but well-meant labor on your behalf.

It has added to our zeal an additional zest for the duties assigned us, lifted us up from the dull routine of daily labor, and pointed us onward and upward, with an assurance that, for the future, a brighter day will shed her refulgent light on dental science. In an effort to irradiate this light—to disseminate in our profession useful knowledge, and strip from it those robes which empiricism would fain throw around it, we feel that we shall have your hearty co-operation; and, more than this, as you pass out into the busy world, this speciality of surgical science, more important than any other, because five times more needed, will always receive from you a fostering hand, and that support which a just appreciation of its worth demands.

Gentlemen, you have selected for your pursuit in life this particular department of medical and surgical science—you have passed through the initiatory steps—worked onward and upward, dipping at one time into the depths of pathological research, tracing from cause to effect the mysterious agencies which work disease in the system—at another you have raised the veil from nature's great laboratory, and beheld the surpassingly beautiful laws which govern chemical action, and also beheld the manner in which the affinities, by which the healthy organs of the dental economy are kept in order, may be broken up—you have studied man in his most wonderful organization, and can truly say, with Israel's prophet, bard, and king, "Man is fearfully and wonderfully made;" here is the perfection of mechanism, God's wondrous skill shown forth—you have been enabled to see the necessity of duly regarding these mechanical laws, so that the forces which propel this wonderful machine may not be disturbed in their appropriate sphere of action—you have thus threaded labyrinth after labyrinth in the pursuit of knowledge, until in this temple of science you have knocked, and not in vain, for her highest honors.

Think not that all is accomplished. This is *your* commencement—you now, as it were, commence life—you commence to apply those instructions you may here and elsewhere have received—may I not say, that you have just fairly commenced your studies—that what has been so fairly commenced, will, with increasing ardor, be pursued—that you will hereafter test, by the closest scrutiny, every proposition you may have received as truth—cherish no theory in opposition to demonstrable facts, however necessary such theory may be to establish any given axiom in dental science. All speculation which runs contrary to the truths of science is worse than useless; be, therefore, not content with conjecture, but first prove, and then receive any given theory of practice. Apply this rule not only to that which may be to you new, but as time and opportunity offers; thus, also, test that which you have already received. Truth will not suffer by the severest ordeal; but each renewed investigation only makes it the more resplendent, rubbing off only more and more of that error which oft in part dims its brightness.

The foundation principles of dental science we have labored to impress on your minds in such a manner that you would see their force and propriety—a course of study which embraces only two or three years, with one or two full courses in this and similar institutions, cannot go into all the detail of practical knowledge. Our medical colleges do not turn out experienced practitioners; and this is especially applicable as it regards operative surgeons. How seldom do they afford to the student opportunity for the manipulations of surgical practice. True, the advantages of hospital practice is of essential service; but even here the practice is in reality confined to the house physician and members of the Faculty, who prescribe for the sick and perform all the operations in surgery.

Our dental institutions in this particular have made, as we think, one step in advance of this mode of teaching: owing to the vast amount of dental operations required, and the great number who are not able to pay for the same, abundant opportunity can be offered, so that each student shall have not only repeated operations performed in his presence, but he may also perform himself almost every operation required in dental practice. He does thus obtain that thorough practical knowledge which fits him for successful and immediate practice, and without which many of the difficult manipulations of operative surgery, and particularly dental surgery, can never be overcome. The infirmary practice, therefore, of our dental school we view as of vast importance to the student.

This peculiar feature, (if we may so call it,) which belongs as yet to the speciality of dentistry, and which, from the nature of the case, and the cause already alluded to, must always attach itself more to this branch of surgical science than any other—must the more advance this department of the healing art, and open a wide field for the full display of that deep research and inventive genius which has of late added so much to our stock of dental knowledge.

But we allude to this not to draw an invidious comparison between the medical and dental schools, or to show the superior advantages in this respect of the latter; but that we may draw attention more particularly to those fundamental principles of our science, which we regard as first in importance, and on no account to be neglected. We refer to a correct pathological view of disease, based on a thorough knowledge of the anatomy of the dental organs, with those which may suffer by disease of the same, as well as that knowledge of chemistry which may teach us to avoid those remedial agents which may act injuriously, and point out those which may be of service. Any amount of practical knowledge not sustained by this proper basis, cannot sustain the dental practitioner in all the exigencies he may be called on to meet. Without these, he must "prescribe in the dark," operate at random, and often aggravate that disease which it is his duty to cure. However much, therefore, we may rejoice in the opportunity offered for practical knowledge in the infirmary of this institution, yet we would so confine and guard the same, that it may not be in advance of that knowledge on which is based true practice; but, like science and art—true twin sisters of knowledge, messengers of love, comfort, and happiness to man—we would have one sustained by the other, each in its appropriate place—science first pointing out the hidden cause of disease and mode of relief, and art applying the remedy.

This demonstrative application of dental science you have, so far as the nature of the case and your studies would permit, witnessed during your course of study—to you, therefore, practice will not be in all respects a novelty; yet, without continued study, you will often find much to perplex and embarrass you in your professional career; every case you are called on to treat will not be clear and distinct in its peculiar characteristics; long and close observation alone will enable you to decide promptly and surely on that course of practice which may best suit a particular case; our oldest practitioners often take much time to investigate before they decide to what extent they shall operate, and how this shall be performed. This is necessary, if not to ensure success in the operation, to give at least to the patient the less amount of pain, as well as shorten the period of suffering.

Eminence, even in dentistry, is not attained in a few short months, or even years—patience, perseverance, application to study and business, will alone ensure this. Need I add, that these, when properly guided by correct principle, will most surely reach this end? Hunter, Bell, Fox, and all such, were once almost unknown students, plodding their way silently but diligently up the ascent of the hill of science. Many started on this journey with them; they ran well for a season; but ease, luxury, and deceptive pleasure clipped fame, to them, of her wings, and as they stopped on the way to gather the feathers which the wind scattered in every direction, they stumbled and fell; while the others kept straight on, still students more close and assiduous than ever, until when *they* fell, it was with their armor all on, and they had reached a higher ascent than any before them. Trace out the history of such as these, and you will most generally observe that in early life they were not so much distinguished for brilliant talents, for ready, quick, and dazzling powers, as for that patient perseverance which knew no repulse, but labored on, labored ever, and always conquered. The same character works still the same results—man is still the same, truth is still the same, and the laws which operate on both still mould and shape the destiny of all who are brought under their influence.

Castles built in the air are very unsubstantial things. They may do to amuse a dreamy imagination, to waft us in our sleeping moments to elysion fields of delight, and even cause us, for a brief period, to revel in all the pomp and circumstance of imperial splendor—but they suit not the realities of life; the walls are too thin to keep out the cold, and too frail to stand the storms and collisions of real life. However well they might suit the fabled houri of the east, yet for this western world we need something far more substantial. The log-cabin affords far more comfort, and possesses more of that which is tangible on which to build hopes for the future.

Prepare yourselves, therefore, to meet the realities of life—to battle all the obstacles which will be arrayed against your future success—to stem every adverse tide of fortune, and moor your bark safe beyond the reach of that receding tide which is drawn back beneath the swelling surge which rides onward to the shore of life.

You commence life in a profession much abused, and this injury often inflicted by those who should be most active in guarding its escutcheon from dishonor. This is occasioned by the fact, that such do not generally appreciate its importance; they see only the surface, have no taste, talent, or disposition for scientific research; hence, argue themselves into the belief, that a dexterous hand and ready manipulation is all that is necessary. Their interest, reputation, and success, urge them on to that leveling process which would keep forever the profession in ignorance, that they may maintain a respectable standing in the same. They do not wish to be considered behind, in the improvements which are made, therefore claim all as their own, or oppose them as useless. Many such you may find; yet, they are rather *outsiders*, and have never entered the fair temple of dental science, by the regular door, "but hath climbed up some other way." The application might be carried out according to scripture.

But we wish not to be understood as giving credit to the oft repeated and much believed report, that dentistry is the *great* field for empiricism. True, this particular speciality of general surgery might with justice claim excuse for such a condition of things. Where can you find any other department of the healing art, which has been so much and long neglected by that mother, whose duty it was to nurse alike all her offspring. This is the more remarkable, when we observe that the cries for help—help to the afflicted—have been longer and louder than from any other; but she was cast off during a long and dreary age of darkness, and while her parent was in open dalliance with the necromantic art, she was nursed by the barbers. Her parent had scarce shaken off this unholy alliance, ere you find dentistry step forth, neatly shaved, and ready for a fair and honorable race up the hill of science. No public benefactions have strewn her pathway—no magnificent halls have been erected by the public for her advancement—no levied tax for her support; but she has stood forth alone. Step by step has she kept pace with medical and surgical science, until now she claims and receives, not donations for a college, not admission to a hospital, but that respect and consideration which her merit demands. She rears her own temple, and founds her own infirmary, and aims to lay a sure foundation for usefulness. Is it then out of place to defend her from the charge of empiricism. That no law has been thrown around her to protect her from the continued molestations of the ignorant, cannot be laid to her account; and even medicine, thus guarded, fares no better if as well.

Does the charge come from the great mass of the public? They have nothing on which to found such an accusation. As a general rule, they have not been in the habit of employing dentists to attend to their teeth. When they employ, for this purpose, silversmiths, coblers, tailors, and even physicians, dentistry should not be blamed for their failures; yet, such has been the custom, and it is done either to cheapen the operation, or for the want of a better—yet, the want of success is, by them, generally attributed to the insufficiency of dental science; and this is a fine excuse also for the operator. As well might physicians be charged with quackery, when, for the want of skill in a blacksmith, a patient may die, to whom he had administered medicine. Take in all your practitioners of medicine, all who prescribe for the sick, your doctors and doctresses, and array in a solid phalanx the quackery thus perpetrated; make our medical fraternity responsible for all this, and dentistry, with all her empiricism, might stand along side, with head erect, unscathed and unabashed by the comparison. Strip both of these hangers on; these excressences held by so slender a cord, that it is scarce perceptible, yet the fungus is large, and seems at times to hide almost the body to which it is attached, and you will find still that a comparison will detract nothing from dentistry as a science.

Dentistry is distinct as a profession, embracing much it is true which belongs to medicine as a science, yet, she works within her own sphere, and among her enlightened members; quackery can find but little to display itself upon. It is therefore mostly confined without the profession, that is, to those who have never, even by education, or aught else, entitled themselves to any of her honors. The line of demarkation is just as plain and distinct here, as in medicine. True, a medical man may not be able to distinguish it; yet, it does exist, as legible as the noon day sun, to the profession, and it is the duty of physicians to be able to trace it. The public and the dentist may scarce, at times, be able to say who are doctors, so many claim the appellation. Much time might be necessary to hunt the proof and search the record, yet we hold, if one is needed, this should be always done. It is bad enough for the unlearned to be mistaken in this matter, and for such we have some sympathy; but those who have proper opportunity for correct information in this particular, and who run headlong into the trap set by the quack, we can scarce pity.

We do not think it necessary that physicians should be practical dentists. It is better otherwise; but they should be able to determine what are good operations and who are the competent. They should be aware that many who claim to be dentists depend on them—the clergy, the judges, and the colonels of our land—for special recommendation to the public. These should all be good judges of dental operations, or, if not, should be very cautious how they claim such discrimination and recommend the unworthy.

We are glad to observe that many of the above named gentlemen have taken a stand against this method of advancing the pretensions of those who cannot depend on their own merit. Quackery in dentistry, therefore, is unlike what it appears in medicine. In the former, no official act has given the semblance of respectability and dignity to charlatanism. It merely clings as yet to the skirt of her garments—every year relaxing her grasp—ready soon, we hope, to be skaken off forever. Not so in the latter; she is covered with parchment; has wrapped herself in the drapery which is made to elevate and adorn medical science, and she struggles with a death-grip for all the honor and substance of the profession. Look at our weeklies and dailies, and they are filled with the sum and substance of the most barefaced and arrant quackery. Scan these documents well, and you will find a majority set off with the insignia of the profession; M. D.'s turn pill makers, compounders of syrup, elixirs, the great panaceas; and to give still greater effect to their wonderful discoveries, they secure letters patent, throw the charm of secrecy around the little knowledge they possess, look wise, keep *mum*, and trust to a credulous public for a fortune. Notwithstanding all this, and the glass-like structure of their dwelling, we are told, by one high in the profession, that he believes that patents and secrecy are common to and are considered honorable among dentists. We repudiate this imputation, and affirm that no department of medical science has dealt less in patents than the dental, and in nearly all cases, where these have been secured, they relate to the instruments used, and not to the practice itself, or to any particular remedial agent needed for the cure of disease.

Let us not be misunderstood. We have no allegations to make against a profession we so much esteem, and of which we claim to be an humble member—far otherwise; for we would gladly see these blotches removed, and shall do all we can for this happy consummation; but we feel it a duty to vindicate dental surgery from a false charge under which she has so long lain passive. The fact is, she has sought no honors but that of doing good, depending on this for her gradual but sure elevation. Possessing until of late no channel by which she could reach the public, her dissenting voice, if raised at all, could scarce be heard but as the feeble and far off echo drowned by the deep tones of her accusers. Her literature has as yet scarce assumed a separate, distinct, and tangible form; yet the last ten years have added more to her treasury in this respect, than a century previous. Every year more and more brings out and develops her vast resources in this particular. Within this period four or five periodicals have been established, devoted exclusively to the science, and several of our best practical works published.

Dentistry, although in practice, not a unit, yet approaches nearer this than any other department of medical science. The division, however, which exists in practice, is not such as to entirely destroy her unity—the gold and amalgam pluggers agreeing in this particular, that gold is the better article for this purpose. The admission, in this case, placing the mercurial advocates on the wrong side of the house, contending for a mode of practice which they acknowledge to be only at times admissible. But we stop not to argue this question, which is scarce debatable.

The profession you have chosen scarce admits of the varied modes of practice which exist in medicine. Our doses are too large for Homœopathy, too metalic for the steamers; we take too much blood for the Eclectics, and the diseases we treat are too urgent for the slow process of Hydropathy; and, however near we may follow in the footsteps of our venerated parent, yet dental operations, unlike those of general surgery, tell their own tales, and trumpet forth her failures. The dead utter no complaints, but carry to the grave all want of skill and evidence of empiricism.

Fortunately, in the practice of your profession, whatever theory you may adopt as to the cure and nature of dental disease, the mode of treatment must be much the same. Whatever speculation may do in the one, will be remedied by that practical experience which has thus far guided, and, it is hoped, will always continue to guide our most worthy practitioners.

We know that certain operations in dentistry, when well performed, and under favorable circumstances, will always arrest the disease and preserve the teeth. This is a good starting point in your practice; so far you have an assurance that you can essentially benefit your patient, and this condition of things exists in a large majority of cases where your services will be required. There is no need, therefore, that you should in these cases deviate from that mode of practice which has been well tested, and is now by our regular dentists so universally adopted: your only difficulty will be how, at times, to put that practice into operation. The time has not yet gone by when many will require to be done that which you know to be wrong. It will be your place not only to operate, but select your own instruments, and choose the manner in which an operation shall be performed. Recollect that the responsibility rests upon you; that failures are now generally attributed to want of skill; that the *dictum* of your patient is never taken into the account, and scarcely ever remembered in the end. As well might a physician undertake to manage a difficult disease, and be guided by the prescriptions of his patient, as for you to expect success when controlled contrary to your judgment in an operation.

That which is well done always remunerates your patient for the time consumed and money expended. This is so applicable to the practice of dental surgery, that it should be remembered as a standing maxim. If it be necessary that an entire day's labor be spent on one tooth for its preservation, and it is important to your patient, it is worth all this, and far more. You will often find that what may at first appear as an easy operation, will require hours of close labor and ingenious application. Let not, however, difficulties discourage your efforts; for every obstacle overcome renders you more perfect in your profession, and better prepares you for the further discharge of your duties. You will find much to try your patience, and the utmost skill you can acquire, and nothing will more test your powers of endurance than an irritable patient.

It will be well to remember that this is, to some extent, constitutional; that all cannot alike control their nervous system; that, indeed, the sensibility in some is higher wrought, than in others; and, hence, that pain as well as pleasure, produces a deeper and more powerful impression. Both may alike desire to aford you every facility for the proper discharge of your duty, yet, are not alike able; and nothing will tend to brace up your pained, distressed, and suffering patient, more than a kind, but firm and gentle manner. Give them your sympathy, gain their confidence, and we know they will do all in their power.

To your professional brethren, who are brethren indeed, be courteous and obliging. We are banded together for a higher and more noble purpose than the mere emoluments of practice. This should be incidental, important to supply our wants, but nothing in comparison to the good we may accomplish. As true as the science of dentistry is progressive, so surely will the public expect at your hands an increased benefit from your services. Let not, then, this expectation meet with disappointment. Remember that success is never far behind our aspirations, when sustained by energetic perseverance, and guided by enlightened reason; determine to excel, and if the summit of your ambitious views are not attained, you will at least place yourselves among the excellent of the earth, and the memory of your worth be embalmed in the recollections of posterity.

Gentlemen—that connection which has existed for the winter, is about to close. You go forth (we have no doubt) buoyant with hope, and desirous of forming a reputation for usefulness, which shall last for life, and be a consolation in death. Regard, then, your services as important to the health, comfort, and life of your patient. Let all your operations have in view this object; continue to cultivate the science you have chosen for a pursuit—one avocation, well pursued, is enough for any man.

That which you have already acquired here, as well as elsewhere, stands as a history of the past, and time, in her rapid flight, waits not to see how it is applied.

Onward moves the course of events—the eddying whirl of man's cares and troubles, makes a few revolutions in the whirlpool of his existence—then sinks in the gulf of forgetfulness. Not so, however, the light of science, for every revolution made on her axis of truth, displays a brighter and brighter effulgence. The dark drapery of ignorance and superstition melts, as her radiant light penetrates the mysteries of nature, until disrobed of error, she stands forth the mistress of our affections. Let me assure you, that this mistress deals not in any of the arts of the coquette. The assiduous wooer, however humble he may be, is always rewarded with something more substantial, than her smiles—she confers her favor, and yields her richest treasure. Gentlemen, we appeal to your decision—has she ever failed to reciprocate your attention, and reward your labor? Has she ever lured you on to a confession, then showed surprise at your declaration? Your answer must be, no. Then yield her not a lukewarm affection; but let every energy, every talent, be put forth to maintain her rights, and defend her escutcheon from dishonor.

If the prowling, hydra-headed monster of charlatanism rears up his many heads, to mar her beauty, and sully her good name; with the sword of truth, guided by an arm of Hercules, sever them off, and continue to strike until none reappear, and dentistry, as a science, stands forth pre-eminently beautiful—perfect in every part—the admiration of all.

Let the ignorant, let the depraved, and the vile, act the part of the quack—but, oh! never let him who hath drank at the fount of knowledge, who hath ever bathed his temples in the refreshing streams of science, or inhaled the fragrant odor of a clear conscience, stoop to her foul embraces.

Her touch is pollution—her course leads downward and downward—along her path are strewn the victims of her avarice; the shrieks, groans, and agonizing cry of the dying, continually pour fourth her requiem, and tell but too surely her work is destruction. May she never find a resting place in our profession.

